# Heterotopic ossification in mice overexpressing Bmp2 in Tie2+ lineages

**DOI:** 10.1038/s41419-021-04003-0

**Published:** 2021-07-22

**Authors:** Belén Prados, Raquel del Toro, Donal MacGrogan, Paula Gómez-Apiñániz, Tania Papoutsi, Pura Muñoz-Cánoves, Simón Méndez-Ferrer, José Luis de la Pompa

**Affiliations:** 1 Intercellular Signaling in Cardiovascular Development & Disease Laboratory, Centro Nacional de Investigaciones Cardiovasculares (CNIC), Melchor Fernández Almagro 3, 28029 Madrid, Spain; 2 CIBER de Enfermedades Cardiovasculares, Madrid, Spain; 3 Centro Nacional de Investigaciones Cardiovasculares (CNIC), Madrid, Spain; 4 Cardiovascular Physiophatology group, Instituto de Biomedicina de Sevilla-IBIS, (Hospital Universitario Virgen del Rocío/CSIC/Universidad de Sevilla). Manuel Siurot, s/n, 41013 Sevilla, Spain; 5 Tissue Regeneration Laboratory, Centro Nacional de Investigaciones Cardiovasculares (CNIC), Madrid, Spain; 6 Department of Experimental & Health Sciences, Universidad Pompeu Fabra (UPF), ICREA and CIBERNED, Dr. Aiguader 88, Barcelona, Spain; 7 Wellcome Trust-Medical Research Council Cambridge Stem Cell Institute and Department of Haematology, University of Cambridge, and National Health Service Blood and Transplant, Cambridge Biomedical Campus, Cambridge, CB2 0AW UK; 8 National Health Service Blood and Transplant, Cambridge Biomedical Campus, Cambridge, CB2 0PT UK

**Keywords:** Mechanisms of disease, Central control of bone remodelling, Haematopoietic stem cells

## Abstract

Bone morphogenetic protein (Bmp) signaling is critical for organismal development and homeostasis. To elucidate Bmp2 function in the vascular/hematopoietic lineages we generated a new transgenic mouse line in which ectopic Bmp2 expression is controlled by the *Tie2* promoter. *Tie2*^*CRE/+*^*;Bmp2*^*tg/tg*^ mice develop aortic valve dysfunction postnatally, accompanied by pre-calcific lesion formation in valve leaflets. Remarkably, *Tie2*^*CRE/+*^*;Bmp2*^*tg/tg*^ mice develop extensive soft tissue bone formation typical of acquired forms of heterotopic ossification (HO) and genetic bone disorders, such as *Fibrodysplasia Ossificans Progressiva* (FOP). Ectopic ossification in *Tie2*^*CRE/+*^*;Bmp2*^*tg/tg*^ transgenic animals is accompanied by increased bone marrow hematopoietic, fibroblast and osteoblast precursors and circulating pro-inflammatory cells. Transplanting wild-type bone marrow hematopoietic stem cells into lethally irradiated *Tie2*^*CRE/+*^*;Bmp2*^*tg/tg*^ mice significantly delays HO onset but does not prevent it. Moreover, transplanting Bmp2-transgenic bone marrow into wild-type recipients does not result in HO, but hematopoietic progenitors contribute to inflammation and ectopic bone marrow colonization rather than to endochondral ossification. Conversely, aberrant Bmp2 signaling activity is associated with fibroblast accumulation, skeletal muscle fiber damage, and expansion of a Tie2+ fibro-adipogenic precursor cell population, suggesting that ectopic bone derives from a skeletal muscle resident osteoprogenitor cell origin. Thus, *Tie2*^*CRE/+*^*;Bmp2*^*tg/tg*^ mice recapitulate HO pathophysiology, and might represent a useful model to investigate therapies seeking to mitigate disorders associated with aberrant extra-skeletal bone formation.

## Introduction

Bone morphogenetic proteins (BMPs) regulate fundamental processes in development and organismal homeostasis [1]. During canonical Bmp signaling, BMPs ligands bind to BMP type I receptors (BMPRIs), or activin-like kinase (ALK) 2,3, or 6. This complex binds to BMP type II receptor (BMPRII), which phosphorylates BMPRI, which in turn phosphorylates regulatory-Smads (Smad1/5/8). Phosphorylated Smad1/5/8 binds to nuclear Smad4, forming a nuclear complex that accumulates in the nucleus, where it is recruited to transcriptional complexes to mediate BMP-driven gene expression [2]. BMPs were discovered owing to their fundamental role in bone formation and homeostasis [1], and BMP2 is critical for chondrocyte proliferation and endochondral bone maturation, and necessary for bone fracture healing [3, 4].

Heterotopic ossification (HO) is bone formation at extra-skeletal sites, including muscle, tendon, ligament, and other connective tissues, and a complication of injury and surgery [5, 6]. HO occurs through intramembranous and endochondral bone formation, resembling fracture repair processes. Lesions are characterized by immune infiltration in damaged connective tissue, which is eventually replaced by endochondral bone through fibroblast proliferation, mesenchymal condensation, and chondro-osteogenic differentiation [6, 7]. Subsequently, the woven bone gives way to the lamellar bone and marrow stroma, with hematopoietic progenitors, adipocytes, osteoblasts and osteoclasts, while capillary-like vessels give rise to bone marrow (BM) sinusoid-type vessels.

Acquired HO is relatively common but its etiology is poorly understood [6]. In contrast, genetic forms, like *Fibrodysplasia ossificans progressiva* (FOP; OMIM #135100, ORPHA337) are rare, but provide mechanistic insight [8–10]. FOP patients have progressive spontaneous and injury-induced HO resulting in complete mobility loss. FOP is caused by a mutation in the gene encoding the type I ACVR1/ALK2 BMP receptor [11]. The ACVR1-R206H mutant receptor acquires the ability to respond to the TGFß family ligand Activin A [12, 13], and becomes sensitive to other BMPs [14–16]. *ACVR1* mutations alone cannot explain the recurrent “flare-ups” resulting in extra skeletal ossification following trauma, muscular fatigue, or other inflammatory insults, which also trigger acquired forms of HO. The innate immune system [17, 18] and local “niche” soft tissue microenvironment [19, 20] need to be further characterized to help clarify this issue. Moreover, Activin A seems to play a significant role in the initial steps of FOP during immune infiltration subsequent to injury. However, once ectopic bone is fused to the normal bone skeleton, additional canonical BMP ligands may be required to sustain ectopic bone development.

The identification of bone osteoprogenitors has generated considerable interest [21, 22]. Skeletal muscle-resident cells including myoblasts [23, 24], satellite cells [25] or fibroadipogenic progenitors (FAPs) have osteogenic differentiation ability [26, 27]. Hematopoietic progenitors participate in bone formation at sites of tissue inflammation, but are insufficient to initiate this process [28, 29]. Endothelial cells in mice constitutively expressing ACVR1-R206H, transform into mesenchymal cells with progenitor properties, that give rise to ectopic bone [30]. However, lineage tracing using a *Tie2*^*CRE*^ driver line and local transplantation of *Tie2*^*CRE*^-derived endothelial cells into skeletal muscle have excluded the endothelium as a source of ectopic bone formation [31]. Rather, non-endothelial Tie2^+^ resident skeletal muscle stem cells including FAP osteoprogenitors appear to be the principal FOP cells-of-origin [31, 32].

In view of ACVR1 receptor activation complexity, the uncertainty of target osteo-progenitors, and modulating “niche” factors, disease modeling is necessary to better understand both acquired and genetic HO. Bmp2 plays fundamental roles in cardiac valves formation and heart chamber patterning [33, 34], but its cardiac overexpression causes lethality [35, 36]. To study Bmp2 vascular gain-of-function postnatally, we generated *Tie2*^*CRE/+*^;*Bmp2*^*tg/tg*^ mice, which overexpress Bmp2 in hematopoietic/endothelial lineages. These mice survive birth, develop pre-calcific valve disease and a systemic bone disorder in skeletal muscle and other connective tissues, resulting in severe skeletal deformities whose nature we have investigated.

## Results

### Endothelial Bmp2 overexpression results in valve dysfunction

We previously generated a transgenic mouse line in which *CAG-*driven *Bmp2* expression is activated upon Cre-mediated removal of a *β-Geo*-stop cassette [35] (Supplementary Fig. 1A). We crossed the *CAG*-*Bmp2* allele with *Tie2*^*CRE*^ line, which is active in hematopoietic/endothelial lineages from E7.5 onwards [37]. Vascular GFP reporter expression was observed at E9.5, confirming Cre-mediated recombination (Supplementary Fig. 1B).

**Fig. 1:**
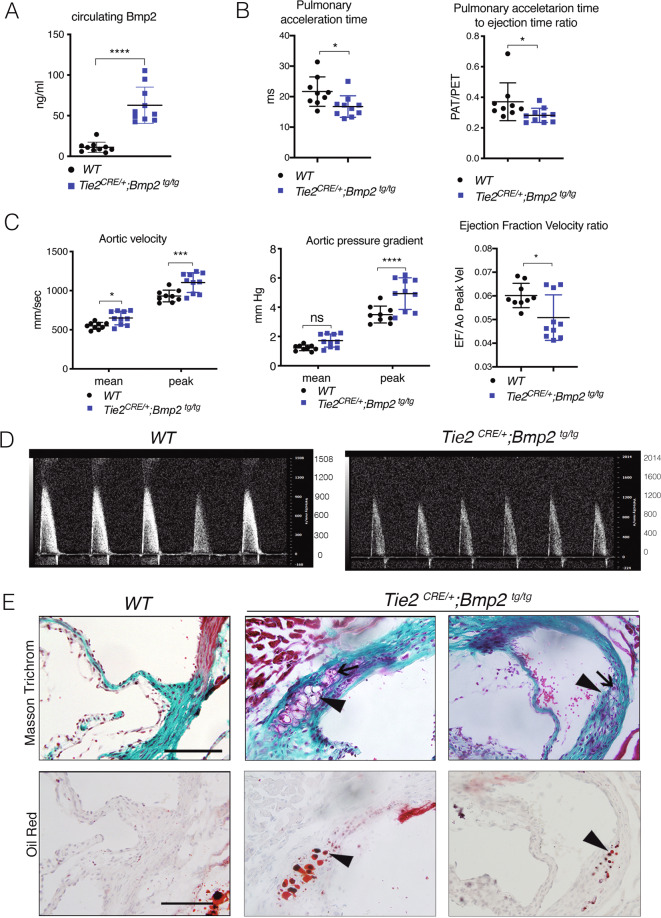
Constitutive endothelial Bmp2 overexpression results in aortic valve dysfunction and pre-calcification. **A** Circulating Bmp2 levels detected by ELISA in WT and Tie2 ^*CRE/+*^*;Bmp2* ^*tg/tg*^ adult mice serum. **B** Quantification of pulmonary acceleration time (PAT, left panel), and PAT-ejection time ratio (PAT/PET, right panel) measured by ultrasound on 16-week-old WT and *Tie2* ^*CRE/+*^*;Bmp2* ^*tg/tg*^ mice. **C** Quantification of the aortic valve velocity (AoV Mean and Peak Vel), and pressure gradient (AoV Mean and Peak Grad) measured by ultrasound. **D** Representative images of acquired data of the aortic velocity peaks detected by ultrasound in WT (≈1000 mm/s) and tg animals (≈1200 mm/s). **E** Top panels: Masson trichromic staining on consecutive sections of aortic valve from 18-week-old WT and *Tie2* ^*CRE/+*^*;Bmp2* ^*tg/tg*^ mice. Chondrocyte island (arrow) in aortic annulus at the base of the leaflet. Bottom panels: Localization of lipid droplets (arrowheads) identified by Oil Red O staining. Unpaired *t* test, two tails, mean ± SD *P < 0.05; ***P < 0.001; ****P < 0.0001; ns, non-significant. Scale bar 200 µm.

Ectopic Bmp2 signaling leads to osteogenic differentiation of valve interstitial cells [38]. To determine the effect of increased endothelial *Bmp2* expression on valve function, we generated *Tie2*^*CRE/+*^*;Bmp2*^*tg/tg*^ mice. At 16 weeks, circulating Bmp2 levels were almost six-fold higher than in WT animals (Fig. 1A). *Tie2*^*CRE/+*^*;Bmp2*^*tg/tg*^ mice showed shortened pulmonary acceleration time and acceleration to ejection time ratio by ultrasound (Fig. 1B), indicating pulmonary hypertension potentially leading to respiratory insufficiency. *Tie2*^*CRE/+*^*;Bmp2*^*tg/tg*^ mice displayed significantly increased aortic valve mean, peak velocity and pressure gradient (Fig. 1C, D). Three of seven animals displayed chondrogenic and lipid droplet islands at the leaflet base (Fig. 1E), indicative of pre-calcific disease. These results indicate that ectopic endothelial and/or hematopoietic Bmp2 expression leads to aortic valve dysfunction compatible with a pre-calcific valve stage.

### Hematopoietic/endothelial Bmp2 overexpression causes a HO

**Fig. 2:**
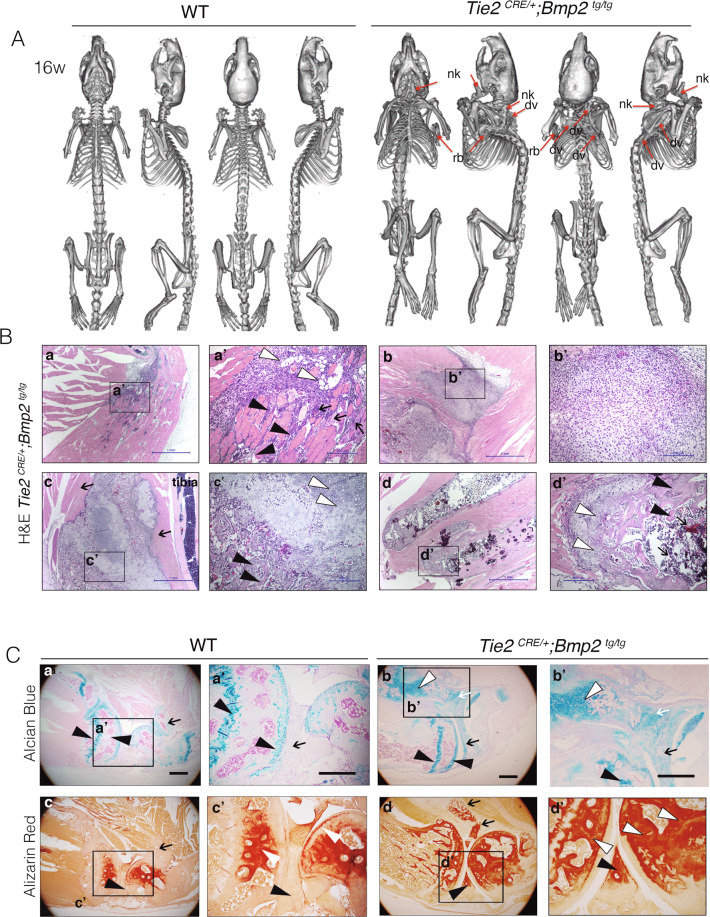
Constitutive Tie2-driven Bmp2 expression causes HO lesions in mice. **A** Nano PET-computed tomography (CT) images of 16-week-old WT and *Tie2* ^*CRE/+*^*;Bmp2* ^*tg/tg*^ mice showing ectopic bone lesions close to ribs, scapulae, and neck (red arrows, ribs, rb; nk, neck; dorsal vertebrae, dv). **B** H&E staining on sections of skeletal muscle of the hindlimbs of *Tie2* ^*CRE/+*^*;Bmp2* ^*tg/tg*^ mice showing histological features typical of HO lesions. **a**. Evidence of inflammation in HO lesion. **a**’ Damaged skeletal muscle fibers with central nuclei (arrows), mononuclear infiltration (black arrowheads), and fat cells (white arrowheads). **b**, **b’** Area of massive fibroblast accumulation. **c** Ectopic bone in skeletal muscle (arrows) next to tibia. **c’** Chondro-osteogenic areas with chondrocytes (white arrowhead), and osteoblasts (black arrowheads). **d**, **d’** Mature ectopic bone with colonizing bone marrow cells (arrows), chondrocytes (white arrowhead), and osteoblasts (black arrowhead). **C** Top panels: Alcian blue staining of sections of WT and *Tie2* ^*CRE/+*^*;Bmp2* ^*tg/tg*^ knee joint. **a**, **a’** In WT, chondrocyte tissue (in blue) is located in the epiphysis region, tip of the bone (black arrowheads), and head of the fibula (arrow). **b**, **b’** In *Tie2* ^*CRE/+*^*;Bmp2* ^*tg/tg*^, chondrocyte tissue is located in the epiphysis region at the tip of the bone (black arrowheads), accumulated in connective tissue (arrow) of the meniscus and head of the fibula (white arrow) and chondrogenic areas (white arrowhead) inside the skeletal muscle. Bottom panels: Alizarin red staining of WT and *Tie2* ^*CRE/+*^*;Bmp2* ^*tg/tg*^ knee joint. **c**, **c’** In WT, osteogenic tissue (in red) is located in the epiphysis region and tip of the bone complementary to chondrogenic areas (white arrowheads). **d**, **d’** Intense staining in the head of *Tie2* ^*CRE/+*^*;Bmp2* ^*tg/tg*^ joints (white arrowheads in (**d**’)). Extra ossification inside the skeletal muscle and head of the fibula (arrows in (**d**)), and the meniscus (black arrowhead). Scale bar 200 µm.

During these studies, we found that *Tie2*^*CRE/+*^*;Bmp2*^*tg/tg*^ mice develop severe scoliosis and ankylosis with depressed locomotor behavior and respiratory insufficiency. Extensive HO was diagnosed by PET-CT at 16 weeks of age and confirmed at autopsy (Fig. 2A; Supplementary Fig. 1C; Supplementary Table 1). Localized HO lesions were observed in 95% (19/20) *Tie2*^*CRE/+*^*;Bmp2*^*tg/tg*^ animals by nano PET-CT (Supplementary Table 1, Supplementary Videos 1–5). Only one of 20 mice remained HO-free (Supplementary Table 1). HO was manifested as fused cervical spine and scapula extensions (47.2%) resulting in severe scoliosis (Fig. 2A; Supplementary Videos 1,2), and as bony plaques adjacent to the chest wall (55%; Supplementary Videos 3). Several animals (65%) displayed bone outgrowths in hindlimbs (Supplementary Table 1; Supplementary Movies 4,5). At 16 weeks, 70% of animals presented HO in multiple areas, while by 20 weeks, 100% presented severe HO lesions extending to most of the areas described (Supplementary Fig. 1C, Supplementary Table 1 and Supplementary Video 6). Of 7 heterozygous *Tie2*^*CRE/+*^*;Bmp2*^*tg/+*^ animals analyzed by nano PET-CT at 16 and 32 weeks, only 2 developed mild lesions by 32 weeks (Supplementary Table 1 and Supplementary Fig. 1D). Circulating Bmp2 levels for heterozygous transgenic mice at 24 weeks was 1.5-fold compared with WT (Supplementary Fig. 1E), while Bmp2 levels of homozygous transgenic *Tie2*^*CRE/+*^*;Bmp2*^*tg/tg*^ animals were 5–6 fold, suggesting that HO is highly sensitive to Bmp2 levels.

HO was associated with typical histopathological changes, including mononuclear cell infiltration (Fig. 2B, a and a’), fibroblast accumulation (Fig. 2B, b and b’), and chondro-osteogenic overgrowths (Fig. 2B, c, c’, d and d’) and development of a BM-like stroma (Fig. 2B, d and d’). Chondrogenic tissue organization in the epiphysis and head of the femur and tibia was similar in *Tie2*^*CRE/+*^*;Bmp2*^*tg/tg*^ and WT animals (Fig. 2C, a, a’, b). However, additional cartilage was present in joints of *Tie2*^*CRE/+*^*;Bmp2*^*tg/tg*^ animals, including osteochondral patches surrounding the tibial head and neck (Fig. 2C, b and b’). Ectopic bone was present adjacent to the normal bone in transgenic animals (Fig. 2C), and bone mass was increased in *Tie2*^*CRE/+*^*;Bmp2*^*tg/tg*^ compared to WT animals (Fig. 2C, c, c’, d and d’). We measured bone mass density (BMD) in tibia and femur bones in WT and *Tie2*^*CRE/+*^*;Bmp2*^*tg/tg*^ animals [39]. BMD was increased about 20% in *Tie2*^*CRE/+*^*;Bmp2*^*tg/tg*^ mice compared to WT (9226 vs. 7720 Hounsfield Units; Supplementary Fig. 2A, B). Ossification of the meniscus (Fig. 2C, d and d’) would likely result in limb immobilization in transgenic animals. Thus, *Tie2*^*CRE/+*^*;Bmp2*^*tg/tg*^ mice show similar histopathological lesions found in human HO.

### Hematopoietic progenitors contribution to HO in *Tie2*^*CRE/+*^*;Bmp2*^*tg/tg*^ mice

**Fig. 3:**
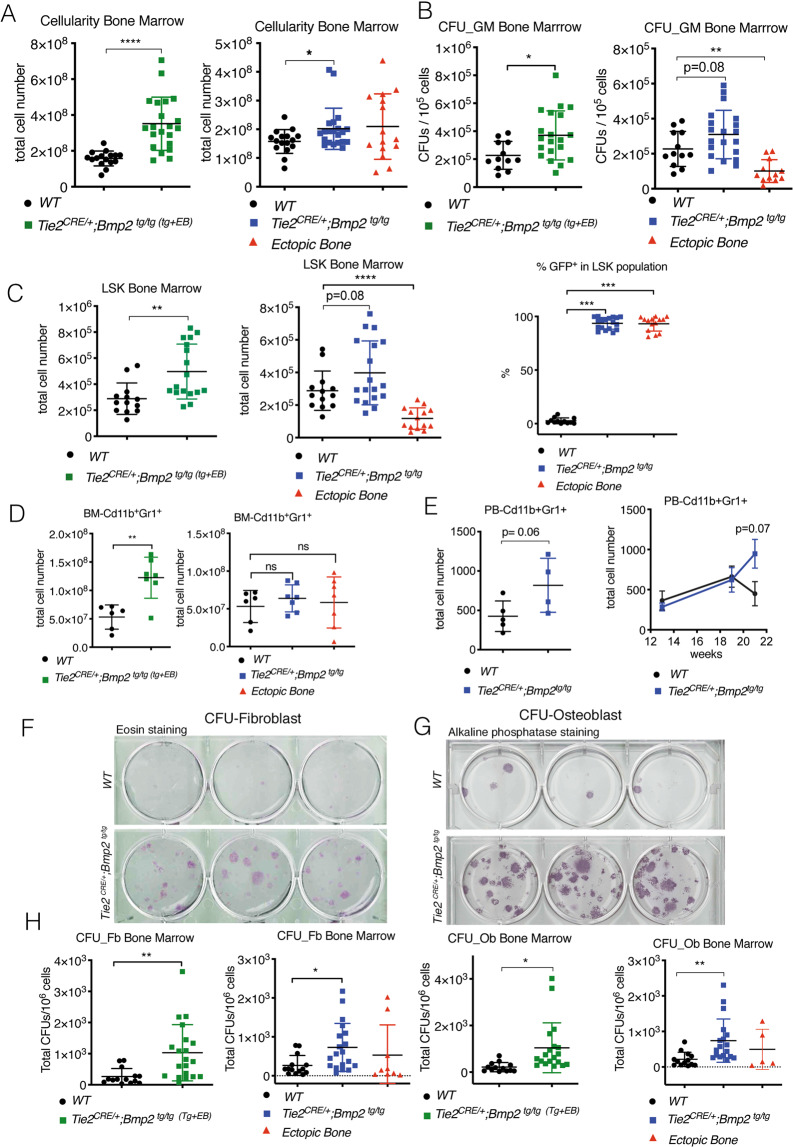
Hematopoietic stem cells contribute to HO in *Tie2*^*CRE/+*^*;Bmp2*^*tg/tg*^ mice. **A** Quantification of bone marrow (BM) cellularity. Left, total BM (combined BM of transgenic (tg) and ectopic bone (EB)) cellularity is increased in *Tie2* ^*CRE/+*^*;Bmp2* ^*tg/tg*^ mice. Right, cellularity of WT BM, tg BM, and ectopic BM. Ectopic BM cellularity is highly variable. **B** Quantification of granulocyte, monocyte colony forming units (CFU_GM). Left, total BM (tg + EB) CFU-GM was increased in *Tie2* ^*CRE/+*^*;Bmp2* ^*tg/tg*^ mice. Right, ectopic BM can give rise to CFU-GMs. **C** Quantification of the FACS-isolated Lin^-^Sca1^+^cKit^+^ (LSK) population. Left, the LSK population is increased in *Tie2* ^*CRE/+*^*;Bmp2* ^*tg/tg*^ BM. Middle, LSK progenitors in ectopic BM are detectable and below normal. Right, the bulk (93.6% in tg and 93.2% in ectopic BM) of the LSK population in *Tie2*^*Cre/+*^*;Bmp2*^*tg/tg*^ BM is GFP^+^. **D** Quantification of the FACS-isolated pro-inflammatory Cd11b^+^Gr1^+^ cells from BM. Left, the Cd11b^+^Gr1^+^ population is increased in total *Tie2* ^*CRE/+*^*;Bmp2* ^*tg/tg*^ BM (tg + EB). Right, varying presence of Cd11b^+^Gr1^+^ cells in ectopic BM. **E**. FACS-isolated pro-inflammatory Cd11b^+^Gr1^+^ cells from PB. Left, Cd11b^+^Gr1^+^ cells are marginally increased in *Tie2* ^*CRE/+*^*;Bmp2* ^*tg/tg*^ PB with HO. Right, Cd11b^+^Gr1^+^ population at different time points, 13, 19, and 21 weeks. At 21 weeks, when mice present HO, pro-inflammatory cells increase in *Tie2* ^*CRE/+*^*;Bmp2* ^*tg/tg*^. **F** Representative eosin staining of CFU-fibroblast (Fb). **G** Representative alkaline phosphatase staining of CFU-osteoblast (Ob). **H** Quantification showing increased CFU-Fb and CFU-Ob in *Tie2* ^*CRE/+*^*;Bmp2* ^*tg/tg*^ BM. **A**, **B**, **C**, **D**, **H**, for WT and *Tie2* ^*CRE/+*^*;Bmp2* ^*tg/tg*^ tg or tg + EB BM groups, unpaired *t* test, two tails, mean ± SD *P < 0.05; **P < 0.01; ***P < 0.001; ****P < 0.0001, ns non-significant. **E** Right panel, two-way ANOVA and Sidak’s correction.

Tie2^+^ cells are potential osteogenic progenitors in HO [21, 30, 31]. To characterize the hematopoietic contribution to HO in *Tie2*^*CRE/+*^*;Bmp2*^*tg/tg*^ mice, bone marrow (BM) was isolated from normal, and ectopic bone of fore- and hindlimbs, scapulae, hips, and sternum of *Tie2*^*CRE/+*^*;Bmp2*^*tg/tg*^ mice, and processed separately. The combined total BM (normal and ectopic bone) cell number was increased in *Tie2*^*CRE/+*^*;Bmp2*^*tg/tg*^ mice (Fig. 3A, left panel), although there were important variations in the total hematopoietic cell number in ectopic bone (Fig. 3A, right panel) due to uneven HO in individual animals.

Granulocyte-monocyte (GM) precursors colony forming-units (CFU) were increased in *Tie2*^*CRE/+*^*;Bmp2*^*tg/tg*^ mice (Fig. 3B), suggesting that enforced Bmp2 expression in Tie2^+^ cells drives hematopoietic progenitor expansion. Interestingly, ectopic bone marrow formed CFU-GM, to a lesser extent than normal WT or tg BM (Fig. 3B, right panel). CFU-GM isolated from transgenic spleen and peripheral blood (PB) were unchanged (Supplementary Fig. 1D).

To support the evidence of increased hematopoietic progenitors in *Tie2*^*CRE/+*^*;Bmp2*^*tg/tg*^ BM, we analyzed Lin^-^Sca1^+^c-Kit^+^ (LSK) cells. This population was increased in total BM (normal and ectopic bones) of *Tie2*^*CRE/+*^*;Bmp2*^*tg/tg*^ mice (Fig. 3C, left panel), and present in ectopic BM (Fig. 3C, middle panel). Consistent with widespread Tie2 expression in hematopoietic progenitors [37], more than 93% of the LSK population in tg normal and ectopic BM was GFP^+^ (Fig. 3C, right panel), reflecting an important contribution of hematopoietic cells to ectopic Bmp2 production and BM formation.

We queried whether inflammation was required for HO in *Tie2*^*CRE/+*^*;Bmp2*^*tg/tg*^ mice. FACS analysis revealed that the pro-inflammatory CD11b^+^Gr1^+^ cell population was increased in combined (normal and ectopic) *Tie2*^*CRE/+*^*;Bmp2*^*tg/tg*^ BM (Fig. 3D, left panel), whereas there was no difference in tg or WT BM (Fig. 3D, right panel). Circulating CD11b^+^Gr1^+^ cells were marginally increased (*p* = 0.06; Fig. 3E, left panel), although their numbers increased in PB (Supplementary Fig. 1E, left panel), indicating that a greater proportion of inflammatory cells are mobilized from *Tie2*^*CRE/+*^*;Bmp2*^*tg/tg*^ BM. We monitored inflammation onset in 4 WT and 4 *Tie2*^*CRE/+*^*;Bmp2*^*tg/tg*^ mice for 7 weeks (at 13, 19, and 21 weeks; Fig. 3E, right panel), and by the time all the *Tie2*^*CRE/+*^*;Bmp2*^*tg/tg*^ mice had developed HO, their inflammatory cell numbers had increased in PB (Supplementary Fig. 3E, right panel).

We tested fibroblast (Fb) and osteoblast (Ob) colony–forming potential in *Tie2*^*CRE/+*^*;Bmp2*^*tg/tg*^ normal and ectopic BM. Fb (Fig. 3F) and Ob (Fig. 3G) CFUs readily formed from WT and transgenic mice BM, from ectopic BM of transgenic animals (Supplementary Fig. 1F). Both CFU-Fbs and CFU-Obs were increased in combined tg BM (normal and ectopic) and tg BM (Fig. 3F, G), suggesting that forced Bmp2 expression in Tie2^+^ cells expands the fibro-osteoblastic BM progenitor population.

BMP2 is known to promote erythropoiesis [40]. By FACS analysis, ProE, EryA, EryC, and erythrocytes (high-Ter119^+^) were all significantly increased in tg mice BM (Supplementary Fig. 1E), suggesting that forced BMP2 expression in Tie2^+^ cells promotes erythropoiesis in vivo.

### Hematopoietic cells overexpressing BMP2 do not trigger HO in wild-type mice

We queried if Tie2^+^ Bmp2-expressing BM hematopoietic cells can give rise to HO in wild-type (WT) mice. Eight-week-old lethally irradiated C57/BL6 WT mice were transplanted with BM hematopoietic stem cells (HSCs) from either WT (*Bmp2*^*tg/+*^) or *Tie2*^*CRE/+*^*;Bmp2*^*tg/+*^ mice (Supplementary Fig. 3A). Hematopoietic cell (CD45^+^) engraftment, was examined by FACS five months after BM transplantation and thereafter bimonthly for over a year. WT mice transplanted with WT HSCs engrafted between 95-100 % of CD45^+^ cells. WT mice transplanted with *Tie2*^*CRE/+;*^*Bmp2*^*t*^^*g/+*^ HSC engrafted 80-90 % of CD45^+^ cells (Supplementary Fig. 3B). Bmp2 increased almost four-fold after 10 months in WT animals transplanted with Bmp2-overexpressing hematopoietic cells (Supplementary Fig. 3C). Hematopoietic-derived Bmp2 accounted for ~50% Bmp2 levels found in *Tie2*^*CRE/+;*^*Bmp2*^*tg/tg*^ mice (compare Fig. 1A and Supplementary Fig. 3B).

We analyzed multi-lineage reconstitution in PB (CD11b^+^, B220^+^ and CD3^+^) by FACS every month for 12 months. Whilst myeloid reconstitution was unaffected, B and T lymphoid cells were decreased at all time points (Supplementary Fig. 3D). BMP2/4 has been shown to antagonize T-cell lineage differentiation [41, 42]. None of the transplanted animals developed HO by Nano-PET-CT (data not shown), even 12 months after the BM transplant assay. Thus, Bmp2 secreted by hematopoietic *Tie2*^*CRE/+*^*;Bmp2*^*tg/+*^ cells in WT mice is not sufficient to drive HO. Alternatively, local expression of other *Tie2-Cre*-targeted cells is crucial to initiate flare-ups and HO.

### Transplanting WT BM into *Tie2*^*CRE/+*^*;Bmp2*^*tg/tg*^ mice delays HO onset

**Fig. 4:**
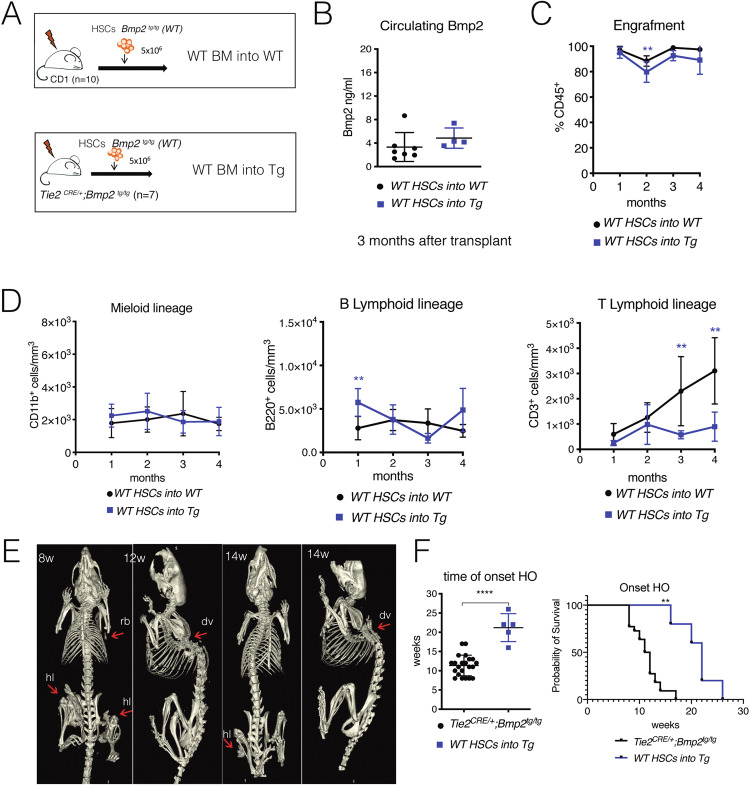
Transplanting WT BM into transgenic *Tie2*^*Cre/+*^*;Bmp2*^*tg/tg*^ mice delays HO onset. **A** Schematic representation of transplant assays. WT (CD1) and *Tie2*^*Cre/+*^*;Bmp2*^*tg/tg*^ mice were transplanted with WT BM cells**. B** Circulating Bmp2 levels are maintained in each group 3 months after transplant. **C** Hematopoietic cell engraftment. FACS-quantification of CD45^+^ cells from PB. *Tie2*^*CRE/+*^*;Bmp2*^*tg/tg*^ and WT mice show similar HSC engraftment. **D** FACS-quantification of myeloid (CD11b^+^), B Lymphoid (B220^+^) and T Lymphoid (CD3^+^) cells in the two groups of transplanted animals. **E** Nano-PET-CT imaging of transgenic *Tie2*^*CRE/+*^*;Bmp2* ^*tg/tg*^ mice transplanted with WT HSC. These animals develop HO (red arrows) in ribs, hind limbs, and dorsal vertebrae (ribs, rb; hind limbs, hl; and dorsal vertebrae, dv) at 8, 12, and 14 weeks after transplant, suggesting that ectopic bones formation started before transplant. **F** Representation of the variability of HO onset in non transplanted *Tie2*^*CRE/+*^*;Bmp2*^*tg/tg*^ mice and *Tie2*^*CRE/+*^*;Bmp2*^*tg/tg*^ transplanted with WT HSCs showing significant delay in the onset of HO (8–18 weeks versus 16-28w). Kaplan-Meier curve showing that *Tie2*^*CRE/+*^*;Bmp2*^*tg/tg*^ mice transplanted with WT HSCs survive 10 weeks longer on average non-transplanted transgenic mice. **C**, **D** Two-way ANOVA and Sidak’s correction **P < 0.01; **F** unpaired *t* test, two tails, mean ± SD. *t* test ****P < 0.0001. **E** Survival curve and survival curve analysis, **P < 0.01.

We asked whether Bmp2 dosage affected HO formation in *Tie2*^*CRE/+*^*;Bmp2*^*tg/tg*^ mice. Eight weeks-old lethally irradiated CD1 mice were used for transplant assays, because *Tie2*^*CRE/+*^*;Bmp2*^*tg/tg*^ mice were in a CD1-enriched mixed background. WT mice were transplanted with 5 million BM nucleated cells from WT (*Bmp2*^*tg/tg*^; *n* = 10) or *Tie2*^*CRE/+*^*;Bmp2*^*tg/tg*^ (*n* = 10) mice (Fig. 4A and Supplementary Fig. 4A). A second group of 8-week-old lethally irradiated *Tie2*^*CRE/+*^*;Bmp2*^*tg/tg*^ mice (*n* = 7) was transplanted with WT (*Bmp2*^*tg/tg*^) HSCs in CD1-enriched mixed background (Fig. 4A). Three transplanted WT mice died after 19 days, and could not be analyzed further. In four, GFP was either not expressed or expressed in 15–20% of total HSCs (Supplementary Fig. 4C, left panel) and excluded. Three surviving mice expressed GFP (Supplementary Fig. 3C, right panel). Circulating Bmp2 levels in two of these mice were comparable to those found in *Tie2*^*CRE/+*^*;Bmp2*^*tg/tg*^ mice (5.9-fold increased) (Supplementary Fig. 4B). Transgenic mice transplanted with WT HSCs had comparable Bmp2 levels to controls (Fig. 4B), confirming the hematopoietic origin of circulating Bmp2.

To determine lineage contributions to BM reconstitution, blood samples were analyzed by FACS monthly over five months following transplantation. CD45^+^ cell engraftment in control and WT groups transplanted with *Tie2*^*CRE/+*^*;Bmp2*^*tg/tg*^ HSCs (*n* = 3) was comparable, reaching 90-98% (Supplementary Fig. 4C). *Tie2*^*CRE/+*^*;Bmp2*^*tg/tg*^ animals transplanted with WT HSCs reached 80–95% engraftment (Fig. 4C). Myeloid CD11b^+^ cell engraftment was similar between groups (Supplementary Fig. 4D and Fig. 4D, left panel). Lymphoid B220^+^ cells increased during the first month compared to controls (Supplementary Fig. 4D, and Fig. 4D, middle panel). In contrast, CD3^+^ T lymphoid cells were not reconstituted to WT levels when transplanted with *Tie2*^*CRE/+*^*;Bmp2*^*tg/tg*^ HSCs (Supplementary Fig. 4D, right panel), as observed previously (Supplementary Fig. 3B). *Tie2*^*CRE/+*^*;Bmp2*^*tg/tg*^ animals transplanted with WT HSCs did not recover normal CD3^+^ levels compared to controls (Fig. 4D, right panel). Thus, B Lymphoid cells were initially depleted in *Tie2* ^*CRE/+*^*;Bmp2* ^*tg/tg*^ mice transplanted with WT HSC but recovered, while T cells remained depleted for the entire duration of the experiment.

*Tie2*^*CRE/+*^*;Bmp2*^*tg/tg*^ mice transplanted with WT hematopoietic cells eventually develop HO, likely because HO was already taking place at the time of transplantation (8 weeks). Five animals presented typical HO with variable severity, including complete hindlimb immobilization by 16 weeks (*n* = 1), dorsal vertebrae at 20 weeks (*n* = 1), and hindlimb and dorsal vertebrae at 22 weeks (*n* = 2) (Fig. 4E). One mouse presented a fat cyst dorsally but no HO, and another developed HO in dorsal vertebrae at 26 weeks (not shown). One mouse remained asymptomatic 37 weeks after transplant. Thus, 5 out of 7 *Tie2*^*CRE/+*^*;Bmp2*^*tg/tg*^ mice reconstituted with WT hematopoietic cells developed HO, with delayed onset compared to non-transplanted animals. Disease onset in *Tie2*^*CRE/+*^*;Bmp2*^*tg/tg*^ mice occurred at 16–28 weeks in transplanted mice, versus 8–18 weeks in non-transplanted ones (Fig. 4F, left panel). *Tie2*^*CRE/+*^*;Bmp2*^*tg/tg*^ mice transplanted with WT HSCs survive 10 weeks longer than non-transplanted transgenics (Fig. 4F, right panel). Therefore, Bmp2 expression in non-hematopoietic cells is essential for HO development, and concomitant Bmp2 expression by hematopoietic cells accelerates this process.

### Chondro-osteogenic differentiation is associated with BMP signaling activation in FAP cells

**Fig. 5:**
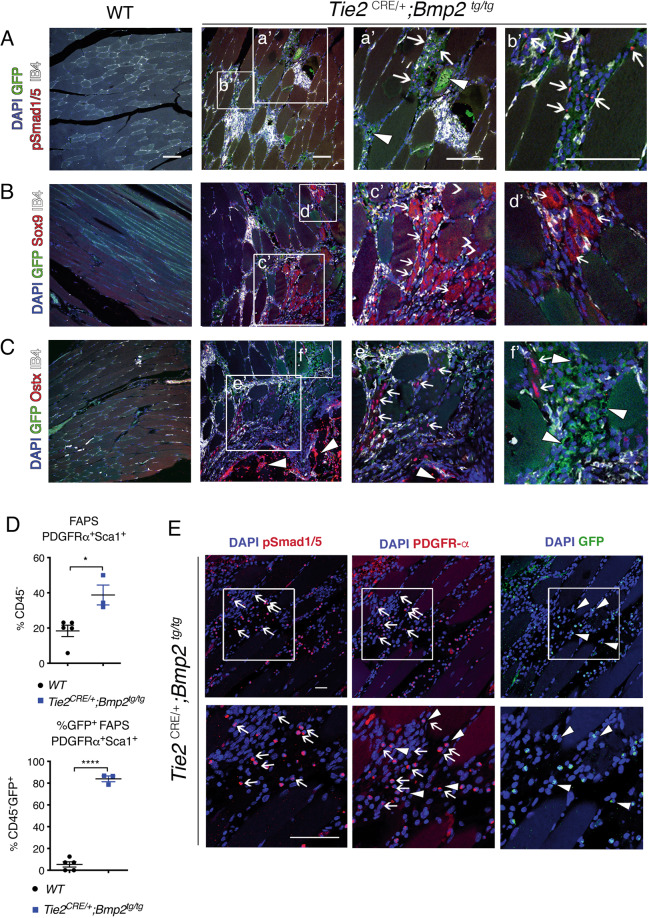
*Tie2*^*Cre/+*^*;Bmp2*^*tg/tg*^ resident skeletal muscle cells are characterized by pSmad1/5 and chondro-osteogenic marker expression, and increased fibro-adipogenic progenitors. Immunodetection of indicated osteo-chondrogenic marker proteins (red), with GFP (green), IB4 (white) and DAPI (blue), on consecutive WT or *Tie2* ^*CRE/+*^*;Bmp2* ^*tg/tg*^ hindlimb skeletal muscle sections, **A** Nuclear immunostaining of GFP^+^ pSmad 1/5 (arrows) interspersed in muscle interstitium (**a’** and **b’)**. White arrowheads indicate GFP^+^ adipocytes. **B** Cytoplasmic Sox9 immunostaining in cells (arrows in **c’** and **d’**) surrounded by damaged skeletal muscle fibers identified as central nuclei (open arrowheads in **c’**). **C**. Nuclear immunostaining of Osterix (Osx) in cells surrounded by fibers (arrows in **e’** and **f’**). Arrowheads indicate strong Osx immunostaining in ectopic bone emerging areas. Arrowheads in **f’** indicate GFP^+^ adipocytes. **D** Top, FACS quantification of the CD45^-^PDGFRα^+^Sca1^+^ fibro-adipogenic (FAP) population in *Tie2* ^*CRE/+*^*;Bmp2* ^*tg/tg*^ skeletal muscle. Bottom, FACS quantification showing that 84% of the CD45^-^GFP^+^ cells are PDGFRα^+^Sca1^+^ cells. **E** Immunostainings of pSmad1/5/8 and PDGFRα (red), GFP (green), and DAPI (blue), on consecutive *Tie2* ^*CRE/+*^*;Bmp2* ^*tg/tg*^ hindlimb skeletal muscle sections showing expression in connective tissue surrounding muscle fibers. Bottom panels are magnifications of areas indicated in top panels. Arrows indicate co-localization of pSmad1/5 and PDGFRα cells. Arrowheads indicate co-localization of PDGFRα and GFP^+^ cells. Cells expressing the three markers are indicated by arrows and arrowheads (middle bottom panel). Scale bars 200 µm. Unpaired *t* test, two tails, mean ± SD *P < 0.05; ****P < 0.0001.

Tie2 marks a subset of resident skeletal muscle cells [31], which potentially contribute to HO. *Tie2*^*CRE/+*^*;Bmp2*^*tg/tg*^ cartilage and bone lesions showed GFP^+^ co-staining with IB4^+^ (Fig. 5A), co-localizing with Tie2^+^ fibroblastic/adipocytic skeletal-muscle cells (Supplementary Fig. 5A, white arrowheads). GFP^+^ infiltrating inflammatory cells expressing IB4 were detected in fibroproliferative areas (Supplementary Fig. 5A), and have been previously described [43]. GFP-expressing fat cells were identified based on their peripheral nuclei (Supplementary Fig. 5B, b’). Phospho-Smad1/5-stained nuclei were detected in scattered cells in fibroproliferative areas (Fig. 5A, a’ and b’ and Supplementary Fig. 5B, a’), as well as among fat cells (Supplementary Fig. 5B, b’). Bmp2 activation may be non-cell autonomous because p-Smad-positive cells did not express GFP (Fig. 5A, a’ and b’ and Supplementary Fig. 5B, a’).

The chondrogenic marker Sox9 was expressed in ectopic bone chondrocyte nuclei (Supplementary Fig. 5C, left panel). Scattered Sox9^+^ nuclei were also found in GFP-expressing fibroblasts (Supplementary Fig. 5C, d’) and damaged skeletal muscle fiber cytoplasm identified by central nuclei (Fig. 5B, c’ and d’, and Supplementary Fig. 5C, c’). This expression pattern was also observed in areas next to HO, although involving very few nuclei (Supplementary Fig. 5C, c’, open arrowheads). The osteogenic marker Osterix, was also detected amongst Sox9-expressing cells in consecutive sections (Fig. 5C, e’, and f’) and in ectopic bone osteoblasts (Supplementary Fig. 5D, arrowheads). Moreover, Osterix staining was observed in larger central nuclei of damaged fibers next to ectopic bone (Supplementary Fig. 5D, e’, and f’).

The expression of chondro-osteogenic markers in damaged skeletal muscle fibers adjacent to ectopic bone, suggests an active repair process. The number of satellite cells labeled by CD45^+^Sca1^-^CD34^+^α7int^+^ was not significantly different between *Tie2*^*CRE/+*^*;Bmp2*^*tg/tg*^ and WT hindlimb skeletal muscle (Fig. 5D, left panel). There were no GFP^+^ satellite cells among *Tie2*^*CRE/+*^*;Bmp2*^*tg/tg*^ skeletal muscle cells (Fig. 5D, right panel), suggesting that satellite cells are not overexpressing Bmp2 and probably not directly implicated in HO in *Tie2*^*CRE/+*^*;Bmp2*^*tg/tg*^ mice.

Resident FAPs are CD45^-^Tie2^+^PDGFRα^+^Sca1^+^ have been proposed as HO cells-of-origin [26]. FAPs were readily increased in *Tie2*^*CRE/+*^*;Bmp2*^*tg/tg*^ skeletal muscle (Fig. 5D, top panel), accounting for 84% of the resident Tie2^+^ cells (Fig. 5D, bottom panel). In fact, among fibroproliferative areas, the majority of pSmad1/5-, PDGFRα- (Fig. 5A, a’, b’; 5E, left panels; Supplementary Fig. 5B, a’, b’ and 5E, middle panels), and Sox9-expressing cells were GFP^+^ (Supplementary Fig. 5C, c’, d’ and 5E right panels). In contrast, Sox9-or Ostx- expressing damaged fibers did not express GFP (Fig. 5B c’, d’; 5C e’, f’ and Supplementary Fig. 5C c’; 5D, e’, f’), suggesting that most FAPs are Tie2^+^ cells, and/or that Tie2 expression is silenced during FAP differentiation.

## Discussion

*Tie2*^*CRE/+*^*;Bmp2*^*tg/tg*^ mice develop HO, apparently spontaneously within 4 months. Normal skeletogenesis is not perturbed, with no obvious HO in juvenile mice, while adult mice present a variety of skeletal deformities including scoliosis, and spinal defects resembling lesions found in HO patients. *Tie2*^*CRE/+*^*;Bmp2*^*tg/tg*^ mice may allow for further studies of therapies designed to mitigate the effects of HO.

Bmp2 is a key pathway activated and causally linked to calcific aortic valve disease in animal models [38, 44–46]. *Tie2*^*CRE/+*^*;Bmp2*^*tg/tg*^ mice present pulmonary valve hypertension and pre-calcific aortic valve dysfunction by 16–18 weeks, characterized by fibrosis, lipid deposition and chondrogenesis. Bmp2 overexpression promotes ectopic EMT in developing *Nkx2.5*^*CRE/+*^*;Bmp2*^*tg/+*^ embryos [35], suggesting that EMT might contribute to pro-calcific disease in adults. However, there is lack of evidence for EMT contributing to adult valve disease pathology [47], and further studies are required. *Tie2*^*CRE/+*^*;Bmp2*^*tg/tg*^ mice develop systemic HO between 8–18 weeks precluding a meaningful study of valve changes, but the effect of ageing over a longer-period might be assessed in *Tie2*^*CRE/+*^*;Bmp2*^*tg/+*^ heterozygotes, which are healthy up to 32 weeks.

Our transplant studies indicate that while Bmp2 transgenic progenitors contribute BM stem cell components, the HO chondro-osteogenic stem/progenitor cell is not BM-derived, consistent with previous studies [28, 29]. Otherwise, hematopoietic/endothelial Bmp2 overexpression profoundly affects hematopoiesis. LSK stem cells, myeloid CFU-GM and Cd11b/Gr11 cells, and fibroblast and osteoblast progenitors are all expanded in transgenic mice. *Tie2*^*CRE/+*^*;Bmp2*^*tg/tg*^ mice display increased Cd11b/Gr11 cells in BM and PB, and increased erythroid lineage differentiation, consistent with in vitro findings [40]. Overall increased BM cellularity is consistent with HSC niche enlargement, enhancement of HSC self-renewal and pool size. Within the BM niche, non-canonical BMP signaling regulates intrinsic HSC maintenance in vivo [48–50], and is implicated in determining the HSC fate, by promoting a pro-lymphoid transcriptional program and sustaining lymphoid-biased HSC commitment [48, 49].

The CD45^-^PDGFRα^+^Sca1^+^ population expansion in skeletal muscle identifies FAPs as potential cells of origin in *Tie2*^*CRE/+*^*;Bmp2*^*tg/tg*^ mice. Evidence for a soft tissue chondro-osteoprogenitor “niche” in damaged skeletal muscle of *Tie2*^*CRE/+*^*;Bmp2*^*tg/tg*^ mice is suggested by the presence of central nuclei, co-immunostaining of isolectin B4 (also a leukocyte marker), pSmad1/5, Sox9, and osterix, in chondro-osteogenic areas of differentiation. Tie2^+^ FAP osteoprogenitors are identifiable by PDGFRα and Sca1 cell surface expression [32], bipotent fibro/adipogenic potential [26, 27], and contribution to ectopic cartilage and bone [31, 32]. HO lesions in *Tie2*^*CRE/+*^*;Bmp2*^*tg/tg*^ mice might be attributed to local Bmp2 release through autocrine/paracrine mechanisms. Conversely, resident skeletal muscle satellite cells (CD45^−^CD34^+^α7int^+^) that give rise to differentiated myocytes [51] were unchanged, suggesting that muscle regenerative potential is not altered. Further studies are required to characterize other progenitor populations residing in skeletal muscle, ligaments and tendons [31, 52] in the context of Bmp2-driven HO.

Previous BMP overexpression studies using a variety of promoters failed to cause HO (reviewed in [53]), because the relevant progenitor cell type had not been targeted. One exception is the transgenic mouse overexpressing BMP4 under control of neuron-specific enolase (Nse) promoter [53, 54]. Our *Tie2*^*CRE/+*^*;Bmp2*^*tg/tg*^ mice resembles this model, matching a stereotyped spreading pattern of HO formation. Neither the NSE-BMP4 model, nor our *Tie2*^*CRE/+*^*;Bmp2*^*tg/tg*^ model recapitulate any of the congenital phenotypes associated with FOP. Moreover, direct versus indirect Bmp effects on target stem/progenitor cell populations cannot be assessed using these models. Nevertheless, *Tie2*^*CRE/+*^*;Bmp2*^*tg/+*^ mice are viable, and develop HO within weeks. This HO model does not require surgical procedures involving the implantation of a BMP-loaded matrix and Bmp2 dosage can be modulated via copy number gene expression. *Tie2*^*CRE/+*^*;Bmp2*^*tg/tg*^ mice are maintained on a heterozygote background, so that a single cross allows for the generation of experimental animals.

## Materials and methods

### Mouse strains and genotyping

The following mouse strains were used: male and female mixed background C56BL/6-CD1 *R26CAGBmp2*^*tg*^ [35] and *Tie2*^*CRE*^ [37]. For simplicity, *R26CAGBmp2*^*tg/+*^ and *R26CAGBmp2*^*tg/tg*^ are abbreviated in the text and figures as *Bmp2*^*tg/+*^ and *Bmp2*^*tg/tg*^, respectively. Details of genotyping will be provided upon request. Recipient transplanted animals were WT C56BL/6, CD1 or *Tie2*^*Cre/+*^*;Bmp2*^*tg/+*^ animals. Animal studies were approved by the CNIC Animal Experimentation Ethics Committee and by the Community of Madrid (Ref. PROEX 83.8/20). Animal procedures conformed to EU Directive 2010/63EU and Recommendation 2007/526/EC regarding the protection of animals used for experimental and scientific purposes, enforced in Spanish law under Real Decreto 53/2013.

### ELISA

Blood samples were taken by submandibular vein puncture from different groups: control WT and *Tie2*^*Cre/+*^*;Bmp2*^*tg/tg*^ mice at 16 weeks of age (n = 10/each group); control WT and heterozygous *Tie2*^*Cre/+*^*;Bmp2*^*tg/+*^ at 24 weeks of age (*n* = 6/each group). 1 year after transplant, WT animals transplanted with WT BMCs, *n* = 6, and WT animals transplanted with *Tie2*^*Cre/+*^*;Bmp2*^*tg/+*^ BMCs, *n* = 8; 3 months after transplant WT animals transplanted with WT BMCs (*n* = 7) and *Tie2*^*Cre/+*^*;Bmp2*^*tg/+*^ animals transplanted with WT BMCs (*n* = 4) were analyzed. Serum was obtained by centrifugation at 4000 rpms for 10 min at RT. Circulating Bmp2 was measured by using the human BMP2 ELISA construction kit (Antigenix America Inc. RHF913CKC) under the manufacturer´s instructions.

### Ultrasound

Mice were anaesthetized by inhalation of isoflurane and oxygen (1.25% and 98.75% respectively) and examined by a 30 MHz transthoracic echocardiography probe. Images were obtained with VEVO 2100 (VisualSonics, Toronto, Canada) from *Tie2*^*Cre/+*^*;Bmp2*^*tg/tg*^ (*n* = 10) and WT (*n* = 9) littermates. Short axis and long axis, B Mode, and 2D M-Mode views were obtained from the M mode by an expert in ultrasound in a blind fashion as described previously [55]. From these images, left ventricle (LV) function was estimated by fractional shortening (FS) and ejection fraction (EF). For FS measurements a long or short-axis view of the heart was selected to obtain an M mode registration in a line perpendicular to the LV septum and posterior wall at the level of the mitral chordae tendinea. Pulmonary acceleration time (PAT) and ejection time (PET) were measured in the parasternal short-axis view by pulsed-wave Doppler of pulmonary artery flow [56]. B-mode and color-Doppler guided pulsed-wave Doppler was used to record the maximal transvalvular jet velocity. Specifically, to avoid Doppler misalignment, coaxial interrogation of the aortic flow was ensured by the operator, and all the measurements were obtained using an angle of interrogation <30°. To correct for flow dependence, we computed an EF velocity ratio (EFVR = EF (%)/maximal aortic velocity [m/s]) as an additional indicator of disease severity [57].

### Positron emission tomography-computed tomography (PET/CT) imaging

In vivo CT imaging was performed on a nanoPET/CT small animal system (Mediso, Hungary) equipped with a micro-focus X-ray source and a high-resolution radiation-imaging device featuring a 1024 × 3596 pixel photodiode array. 16-week-old *Tie2*^*Cre/+*^*;Bmp2*^*tg/tg*^ (*n* = 12) and WT (*n* = 10), or 20 week-old *Tie2*^*Cre/+*^*;Bmp2*^*tg/tg*^ (*n* = 5); 16 and 32-week-old *Tie2*^*Cre/+*^*;Bmp2*^*tg/+*^ (*n* = 7) and WT (*n* = 6) or *Tie2*^*Cre/+*^*;Bmp2*^*tg/tg*^ transplanted with WT BMCs (16 (*n* = 1), 20 (*n* = 1), 22 (*n* = 2) and 26 (*n* = 1) weeks were analyzed. The mice were anesthetized using isoflurane 2% and 1.8 L/min oxygen flow, and positioned in a thermo regulated (38.7 °C) mouse bed with an ophthalmic gel in their eyes to prevent retinal drying. The scan parameters used for the CT measurements were an X-ray beam current of 145 µA and a tube voltage of 55 kVp. Acquisitions and reconstruction were performed by an expert in PET/CT in a blind fashion using proprietary Nucline software (Mediso, Hungary) and analyzed by a whole-body bone 3D volume rendering using OsiriX software (Pixmeo, Switzerland).

### Bone mass spectrometry

Nano-PET-CT acquired and 3D-reconstructed images from 16-week-old WT and *Tie2*^*Cre/+*^*;Bmp2*^*tg/tg*^ animals (*n* = 3 of each group) were further analyzed for bone mass densitometry. Hind limbs mean attenuation coefficient of X rays expressed in Hounsfield Units was measured after segmenting bones using the Multimodality Workstation MMWKS [39].

### Histology

Skeletal muscles and bone tissue (and ectopic bone in transgenic animals) were fixed in 4% PFA for 24 h at 4 °C, and after decalcification with ImmunoCal (StatLab, Fisher scientific) embedded in paraffin or sucrose treated to be cryopreserved in OCT. Hematoxylin/eosin (H&E), Masson´s trichromic, Alizarin red, and Alcian blue stainings were performed according to standard protocols on paraffin-embedded 7 μm sections. Oil Red, staining was performed according to standard protocols on 5 μm cryosections.

### Immunohistochemistry

Paraffin-embedded 7 μm sections of hind limb tissues were citrate-unmasked and stained with the following primary antibodies: polyclonal P-Smad 1/5 (1:100; (41D10) 9516, Cell Signaling Technology), polyclonal Sox9 (1:100, Santa Cruz Biotechnology, sc-20095), polyclonal Sp7/Osterix (1:100, Abcam ab22552), monoclonal GFP living Colors (1:100 Clontech 632381) or polyclonal GFP (1:200; Origene/Acris lab R1091P) and monoclonal CD140a (PDGFRα) (1:100 (APA5) ThermoFisher 17-1401-81). Secondary antibodies were as follows: goat-anti-rabbit HRP (P0448, DakoCytomation; 1:100), goat-anti-mouse HRP (P0447, DakoCytomation; 1:100) goat-anti-rat (ThermoFisher, 31470 1:100) HRP-coupled secondary antibodies and donkey-anti-goat-HRP (ThermoFisher, PA128664). The signal was tyramide signal amplified (TSA) with coupling to Cy3 (NEL744, Perkin Elmer; 1:100) or fluorescein ((NEL744 or NEL741 Perkin Elmer; 1:100)

### Confocal imaging

Confocal images of tissue sections were acquired with a Nikon A1R laser scanning confocal microscope and NIS-Element SD Image Software. Images of stained explants were collected as z-stacks. Z-projections and lateral sections were assembled using ImageJ. Images were processed in Adobe Photoshop Creative Suite 5.1.

### CFU assays

16–20-week-old WT (*n* = 16) and *Tie2*^*Cre/+*^*;Bmp2*^*tg/tg*^ animals (*n* = 20) were sacrificed and blood samples were obtained by cardiac puncture. Spleen and bones were dissected from posterior and anterior limbs, hips, and sternum. Extra bone formations from transgenic animals were dissected and processed separately. BM cells were obtained by crushing bones with a mortar in PBS. The solution containing BM cells was separated, and the remaining bone was treated with Collagenase I for 45 min at 37 °C in a shaking bath to obtain stromal cells. All samples were 70 μm–filtered. Red blood cells were removed from BM and spleen samples using lysis buffer (0.15 M NH4Cl for 10 min at 4 °C) and cell number was determined. Peripheral blood mononuclear cells were isolated from diluted-blood (300 μl of PB mixed with 1.5 ml phenol-red-free RPMI). These samples were added carefully over 2 ml of Lympholite-M (Cedarlane) and were carefully layered on top and centrifuged without brake for 25 min at RT. The halo phase with mononuclear cells was centrifuged at 1500 rpms for 10 min. Cells were resuspended in 200 μl RPMI w/o phenol red. For CFU-GM, cells were mixed with methyl-cellulose and seeded on low adherence p35mm (BM 1 × 10^4^ cells; Spleen 4 × 10^5^ cells, PB 200 μl) by duplicate. The colonies were scored after 7 days in culture. For CFU-Fb and CFU-Ob (WT, *n* = 13 and 14; Tg *n* = 19), cells were plated by triplicate in three wells of six-well plates (1 × 10^6^/well) in α-MEM + 15% FBS. CFUs were stained and counted after a week in culture.

### BM transplantation assays

For BM transplantation assays we used 8-week-old male WT C57BL6 or heterozygous *Tie2*^*Cre/+*^*;Bmp2*^*tg/+/−*^ (mixed background) as donors, (*n* = 2 per transplantation assay) and two groups of male 8-week-old WT as recipients (*n* = 7 and 8 per transplantation assay). Briefly, lethality irradiated receptor mice (11 grays) were transplanted with 2 × 10^6^ bone marrow cells (BMCs) by tail vein injection. BMCs were obtained as mentioned above in FACS analysis section. The homogenized samples were filtered through a 40-μm mesh to obtain single-cell suspensions, and depleted of red blood cells by lysis. For reciprocal transplantation assays we used 8-week-old WT CD1 or *Tie2*^*Cre/+*^*;Bmp2*^*tg/tg*^ as donors, (*n* = 2 per transplantation assay) and two groups of 8-week-old WT CD1 (*n* = 10) or *Tie2*^*Cre/+*^*;Bmp2*^*tg/tg*^ (*n* = 7), as recipients without HO symptoms. In order to check the engraftment of hematopoietic cells in each group of transplants, we bled the animals every month after the transplant, until 4 (WT BMC into *Tie2*^*Cre/+*^*;Bmp2*^*tg/tg*^) or 5 (*Tie2*^*Cre/+*^*;Bmp2*^*tg/tg*^ BMC into WT) months post-transplant.

### Isolation of skeletal muscle cells

Skeletal muscle tissues (tibialis anterior, gastrocnemius, quadriceps) were dissected and minced from hind limbs of wt and *Tie2*^*Cre/+*^*;Bmp2*^*tg/tg*^ 9–11- week-old animals. The minced tissue was digested in Collagenase I (0.25 %), and II (0.2 %) in DMEM/10% FBS for 1 h min at 37 °C in a shaking bath. Cells were washed with DMEM 2 %FBS, 100- and 70-μm filtered, washed with DMEM/2 %FBS, and centrifuged. The pellet was resuspended in PBS/2% FBS to determine cell number, centrifuged, and resuspend in staining buffer (PBS/2%FBS).

### FACS analysis

For BM FACS analysis, 16–20-week-old WT and *Tie2*^*Cre/+*^*;Bmp2*^*tg/tg*^ animals were sacrificed and bones were dissected as described in CFU assays section. 1 × 10^6^ cells were labeled with the following cocktail of antibodies: LSKs (WT, *n* = 13; Tg, *n* = 17): biot-Lin (1:100, BD Biosciences 559971)/St-APC/Cy7 (1:100, BD Biosciences 554063), C-Kit-PE-Cy7 (1:400, BD Biosciences 558163), Sca1-PE (1:200, BD Biosciences cat no. 553336); Erytrhoid lineages (WT, *n* = 4; Tg, *n* = 4) CD71-APC, BD Biosciences 567258); Ter-119-PE-Cy7 (1:100, BD Pharmigen 560509); Pro-inflammatory cells (WT, *n* = 6; Tg, *n* = 7) CD11b-PE (1:200, BD Biosciences 553311); Gr1-APC (1:100, BD Biosciences 553129) and DAPI for viability. The total number of each BM lineage was calculated referred to the total number of cells obtained from BM. Total number of spleen CFUs (WT, *n* = 5; Tg, *n* = 15) and PB-CFUs (WT, *n* = 11; Tg, *n* = 13), PB-pro-inflammatory cells (16–20-week-old mice WT, *n* = 5; Tg, *n* = 4 or the group at 13, 19 or 21 week-old mice WT *n* = 3, tg *n* = 4) was calculated referred to the total number of cells obtained from spleen or white blood cell number determined by hematology. For transplants multi-lineage engraftment FACS analysis peripheral blood cells were labeled with the following cocktail of antibodies: hematopoietic cells CD45-APC (1:100, BD Biosciences 559864), myeloid lineages CD11b-PE (1:200, BD Biosciences 553311), lymphocytes B biot-B220 (1:200, BD Biosciences 559864)/st-APC-Cy7 (1:100), and lymphocytes T CD3-PERCP-Cy5 (1:200, BD Biosciences 560527). For muscle cells FACS analysis cells were labeled with the following cocktail of antibodies: CD45-AV450 (1:200, BD Biosciences 560501), Sca1-PE-Cy7 (1:200, BD Biosciences 558162), α7-Int-PE (1:100, AbLab AB10STMW215), CD34-Alexa647 (1:40, BD Pharmigen 560230), CD140a (PDGFRA) Monoclonal Antibody (APA5), Biotin (1ː200, ThermoFisher 13-1401-82), streptavidin-APC-Cy7 (1:100, BD Biosciences 554063) and 7AAD (1:100, BD Pharmigen 559925) for viability. Satellite cells were identified as CD45^+^Sca1^-^CD34^+^α7int^+^ and fibro-adipogenic cells as CD45^-^PDGFRα^+^Sca1^+^.

### Statistics

Due to the high variability in HO onset and severity, the analysis was made in groups *n* > 10 in some cases. Statistical assessment is indicated in the figure legends. For each experiment comparing two groups, a mean ± SD is represented and a two-tailed *t* test was performed. For experiments comparing two groups at different time points, a mean ± SD is represented at each timepoint and a two-way ANOVA followed by Sidak’s correction was performed. *P < 0.05; **P < 0.01; ***P < 0.001; **** *P* < 0.0001.

## Supplementary information

Suppl. Information

Suppl. Figure 1

Suppl. Figure 2

Suppl. Figure 3

Suppl. Figure 4

Suppl. Figure 5

Suppl. Table 1

Suppl. Video 1.

Suppl. Video 2.

Suppl. Video 3.

Suppl. Video 4.

Suppl. Video 5.

Suppl. Video 6.

## Data Availability

Data sharing is not applicable to this article as no datasets were generated or analyzed during the current study.
