# Supplementary material for: Heterotopic ossification in mice overexpressing Bmp2 in Tie2+ lineages
Source: Cell Death Dis. 2021 Jul 22;12(8):729. doi: 10.1038/s41419-021-04003-0 (PMC8298441; doi:10.1038/s41419-021-04003-0)
Supplement: Supplementary file 1 — Suppl. Information [file 41419_2021_4003_MOESM1_ESM.docx]

SUPPLEMENTARY INFORMATION

**Heterotopic ossification in mice overexpressing Bmp2 in Tie2+ lineages**

Belén Prados, Raquel del Toro, Donal MacGrogan, Paula Gómez-Apiñániz, Tania Papoutsi, Pura Muñoz-Cánoves, Simón Méndez-Ferrer and José Luis de la Pompa

**SUPPLEMENTARY FIGURE LEGENDS**

**Supplemental Figure 1. *Tie2 ^CRE/+^;Bmp2 ^tg/tg^* mice display ectopic limb ossification.**

**A.** Schematic representation of *Tie2^CRE^*-mediated Bmp2 overexpression. The *CAG* cassette floxed *β-Geo*-(STOP) cassette followed by the Bmp2 cDNA and *IRESeGFP* are targeted into the *Rosa26* (R26) locus. Upon *CRE*-mediated recombination *Bmp2-IRESeGFP* is conditionally expressed under the control of CAG and *R26* promoters. **B.** Confocal images of WT and *Tie2 ^Cre/+^;Bmp2 ^tg/tg^* transgenic (tg) hearts at embryonic day (E) 9.5 showing GFP expression in endocardium and endothelium. Abbreviations: avc, atrioventricular canal; v, ventricle; a, atrium. **C.** Nano-PET-CT imaging of WT and transgenic *Tie2^CRE/+^;Bmp2 ^tg/tg^* micel showing HO in forward and hind limbs and knee (red arrows, forward limb, fl; hind limbs, hl; knee, kn,). **D.** Nano-PET-CT imaging at 16 and 32 weeks of two *Tie2^CRE/+^;Bmp2 ^tg/+^* heterozygous mice showing mild HO in dorsal vertebrae, dv (red arrows) only at 32 weeks. **E.** Circulating Bmp2 levels detected by ELISA in 24 week-old WT and *Tie2^CRE/+^;Bmp2 ^tg/+^* mice serum. Unpaired t test, two tails, mean ±SD.

**Supplemental Figure 2. Bone mass density** **and contribution of hematopoietic progenitors to HO.**

**A.** Nano-PET-CT reconstructed images showing tibia and femur bones of WT and *Tie2 ^Cre/+^;Bmp2 ^tg/tg^* mice. **B.** Quantification of the bone mass densitometry showing increased bone mass density on transgenic mice. **C.** Left, quantification of granulocyte, monocyte colony forming units (CFU_GM) as per 10^5^ seeded cells in spleen and right, PB. **D.** Left, increased PB pro-inflammatory cells represented as percentage (%) of viable cells (p=0.013) in transgenic *Tie2^CRE/+^;Bmp2 ^tg/tg^* mice. Rigth, Cd11b^+^Gr1^+^ population at different time points, 13, 19 and 21 weeks. At 21weeks, when mice present HO, pro-inflammatory cells significantly increase in *Tie2 ^CRE/+^;Bmp2 ^tg/tg^*. **E.** Representative eosin staining of CFU-Fibroblast and CFU-osteoblast alkaline-phosphatase (AP) staining, colonies formed by ‘BM’ of ectopic bone mesenchymal cells. **F.** Quantification of erythroid lineages analysed by FACS. Erythroid precursor lineages are globally increased in *Tie2^CRE/+^;Bmp2 ^tg/tg^* BM. ProE, pro-erythroid lineage; Ery A; Erythroid type A lineage; Ery B, Erythroid type B lineage; Ery C, Erythroid type C lineage and Ter119^+^, mature erythrocytes. B, D, and F for each lineage WT vs *Tie2 ^CRE/+^;Bmp2 ^tg/tg^* groups unpaired t test, two tails, mean ±SD **P*< 0.05; ***P* <0.01; ****P* <0.001.

**Supplemental Figure 3. Transplanting heterozygous *Tie2^Cre/+^;Bmp2^tg/+^*** **BM into WT mice depletes the T lymphoid lineage but does not result in HO.**

**A.** Schematic representation of transplants assays. WT mice were transplanted with control WT or *Tie2^Cre/+^;Bmp2^tg/+^* heterozygous HSCs**. B**. Hematopoietic cell engraftment. FACS-isolated CD45^+^ cells from PB. *Tie2 ^CRE/+^;Bmp2 ^tg/+^* HSCs show less efficient engraftment compared to WT cells. **C.** Bmp2 levels increased 3.9-fold 10 months after transplanting *Tie2 ^CRE/+^;Bmp2 ^tg/+^* HSCs into WT mice. **D**. FACS quantification of myeloid (CD11b^+^), B Lymphoid (B220^+^) and T Lymphoid (CD3^+^) cell lineages in the two groups of transplanted mice. The T cell lineage was severely depleted in WT mice transplanted with *Tie2 ^CRE/+^;Bmp2 ^tg/+^* HSCs. C, unpaired t test, two tails, mean ±SD **** *P* <0.001. In B and D two-way ANOVA and Sidak’s correction. **P*< 0.05; ***P* <0.01; ****P* <0.001; **** *P* <0.0001.

**Supplemental Figure 4. Transplanting *Tie2^Cre/+^;Bmp2^tg/tg^*** **BM into WT mice suppresses the T lymphoid lineage but does not result in HO.**

**A.** Schematic representation of the transplant assay. WT mice (n=10) were transplanted with *Tie2^Cre/+^;Bmp2^tg/tg^* BM cells**. B**. Circulating Bmp2 levels in *Tie2 ^CRE/+^;Bmp2 ^tg/+^* -transplanted WT mice (n=2) increase 5.9-fold at 3 months. **C.** Hematopoietic cell engraftment. FACS-isolated CD45^+^ cells from PB. *Tie2 ^CRE/+^;Bmp2 ^tg/tg^* CD45^+^ cells either engraft poorly (n=4; left panel) or display similar engraftment to the control WT group (n=3; right panel). **D**. FACS-isolated myeloid (CD11b^+^), B Lymphoid (B220^+^) and T Lymphoid (CD3^+^) cells in transplanted animals. Left, myeloid lineage is unaffected. Middle, the B cell lineage is initially increased in *Tie2 ^CRE/+^;Bmp2 ^tg/tg^* mice transplanted with WT HSC but becomes normalized during the following months. Right, T cell lineage is reconstituted in WT mice but depleted in *Tie2 ^CRE/+^;Bmp2 ^tg/tg^* mice. C, D, two-way ANOVA and Sidak’s correction **P*< 0.05; ***P* <0.01; ****P* <0.001; **** *P* <0.0001.

**Supplemental Figure 5. *Tie2^CRE+;^Bmp2 ^tg/tg^* resident skeletal muscle cells express pSmad1/5/8, chondrogenic and osteogenic markers.**

Immunodetection of indicated osteo-chondrogenic marker proteins (red), with GFP (green), IB4 (white) and DAPI (blue) on consecutive *Tie2 CRE^/+^;Bmp2 ^tg/tg^* hindlimb skeletal (sk) muscle sections. **A.** GFP immunostaining in fibroproliferative areas. White arrows point endothelial cells expressing both GFP and IB4 surrounding skeletal muscle fibers. White arrowheads indicate GFP non-endothelial cells. Black arrowheads indicate IB4 non-expressing-GFP cells. Open arrowheads indicate central nuclei of damaged fibers. **B.** Nuclear immunostaining of pSmad 1/5/8-positive nuclei (white arrows, **a’** and **b’**), and several pSmad 1/5 and GFP-positive cells (black arrows) interspersed in skeletal muscle interstitium (**a’** and **b’** and GFP/pSmad panel). White arrowheads point to GFP expressing adipocytes. Open arrowheads indicate central nuclei on damaged fibers. **C.** Cytoplasmic Sox9 staining in cells near osteogenic regions (arrows in **c’**) and GFP^+^ cells inside areas of massive accumulation (black arrowheads in **d’** and GFP/Sox9 panel) and central nuclei of damaged skeletal muscle fibers (open arrowheads in **c’**). White arrowheads point to GFP expressing adipocytes. **D.** Osterix (Osx) immunostaining in ectopic bone emerging areas (arrowheads). Nuclear Osx staining in GFP^-^ cells near osteogenic regions­­ containing Sox9 expressing cells (arrows in **e’** and **f’** and GFP/Ostx panel) and central nuclei of damaged skeletal muscle fibers (open arrowheads in **e’**). Scale bar 200 µm. **E.** FACS quantification of the CD45^-^Sca1^+^CD34^+^α7int^+^ satellite population in *Tie2 ^CRE/+^;Bmp2 ^tg/tg^* skeletal muscle. Right, absence of GFP expression in CD45^-^Sca1^-^CD34^+^α7int^+^ cells. Unpaired t test, two tails, mean ±SD.

**SUPPLEMENTARY TABLES**

**Suppl. Table 1.** Summary of PET/CT imaging and histological findings.

**SUPPLEMENTARY VIDEOS**

**Suppl. Video 1.** Axial and limbs skeletal images from 16 weeks-old wild type (control) animal (RLA89).

**Suppl. Video 2.** Axial and limbs skeletal images from 16 weeks-old *Tie2 CRE^/+^;Bmp2 ^tg/tg^* animal (RLA78).

**Suppl. Video 3.** Axial and limbs skeletal images from 16 weeks-old *Tie2 CRE^/+^;Bmp2 ^tg/tg^* animal (RLA79).

**Suppl. Video 4.** Axial and limbs skeletal images from 16 weeks-old *Tie2 CRE^/+^;Bmp2 ^tg/tg^* animal (RLB7). Note HO in hindlimb.

**Suppl. Video 5.** Axial and limbs skeletal images from 16 weeks-old *Tie2 CRE^/+^;Bmp2 ^tg/tg^* animal (RLB8). Note HO in hindlimb.

**Suppl. Video 6.** Axial and limbs skeletal images from 20 weeks-old *Tie2 CRE^/+^;Bmp2 ^tg/tg^* animal (RLB110). Note HO in hindlimb.
