# Supplementary figures and images for: Heterotopic ossification in mice overexpressing Bmp2 in Tie2+ lineages
Source: Cell Death Dis. 2021 Jul 22;12(8):729. doi: 10.1038/s41419-021-04003-0 (PMC8298441; doi:10.1038/s41419-021-04003-0)

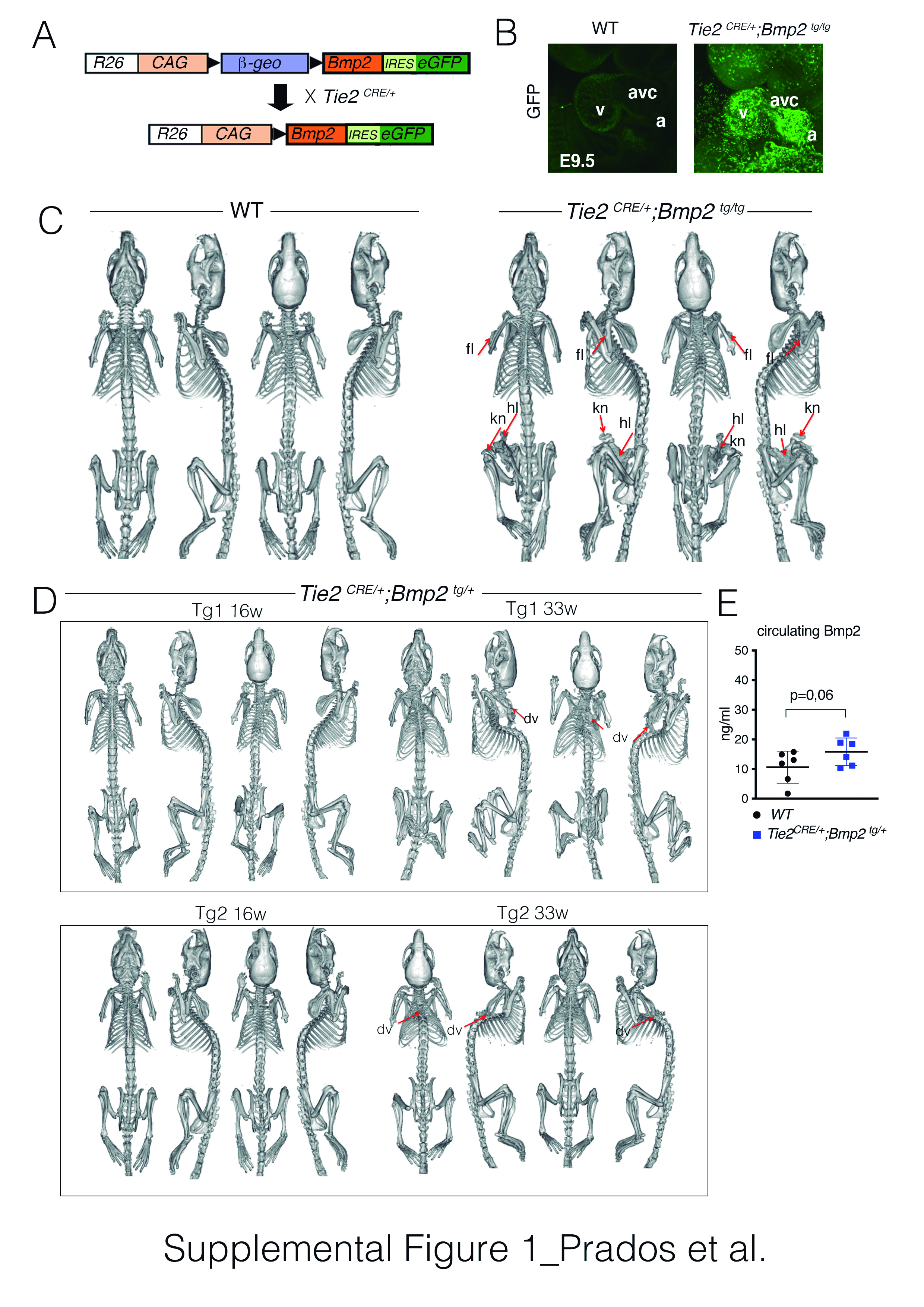

Supplement: Supplementary file 2 — Suppl. Figure 1 [file 41419_2021_4003_MOESM2_ESM.jpg]

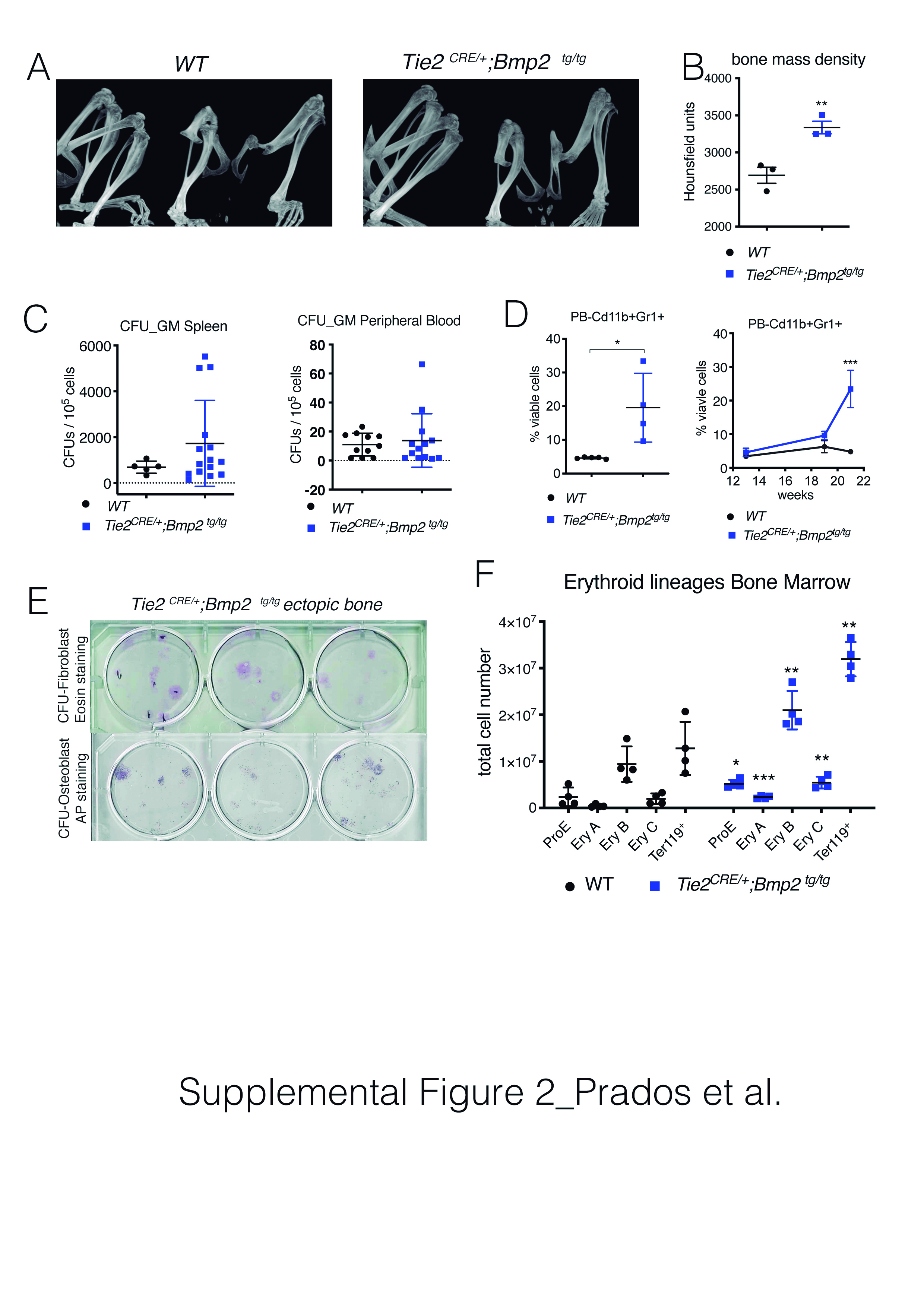

Supplement: Supplementary file 3 — Suppl. Figure 2 [file 41419_2021_4003_MOESM3_ESM.jpg]

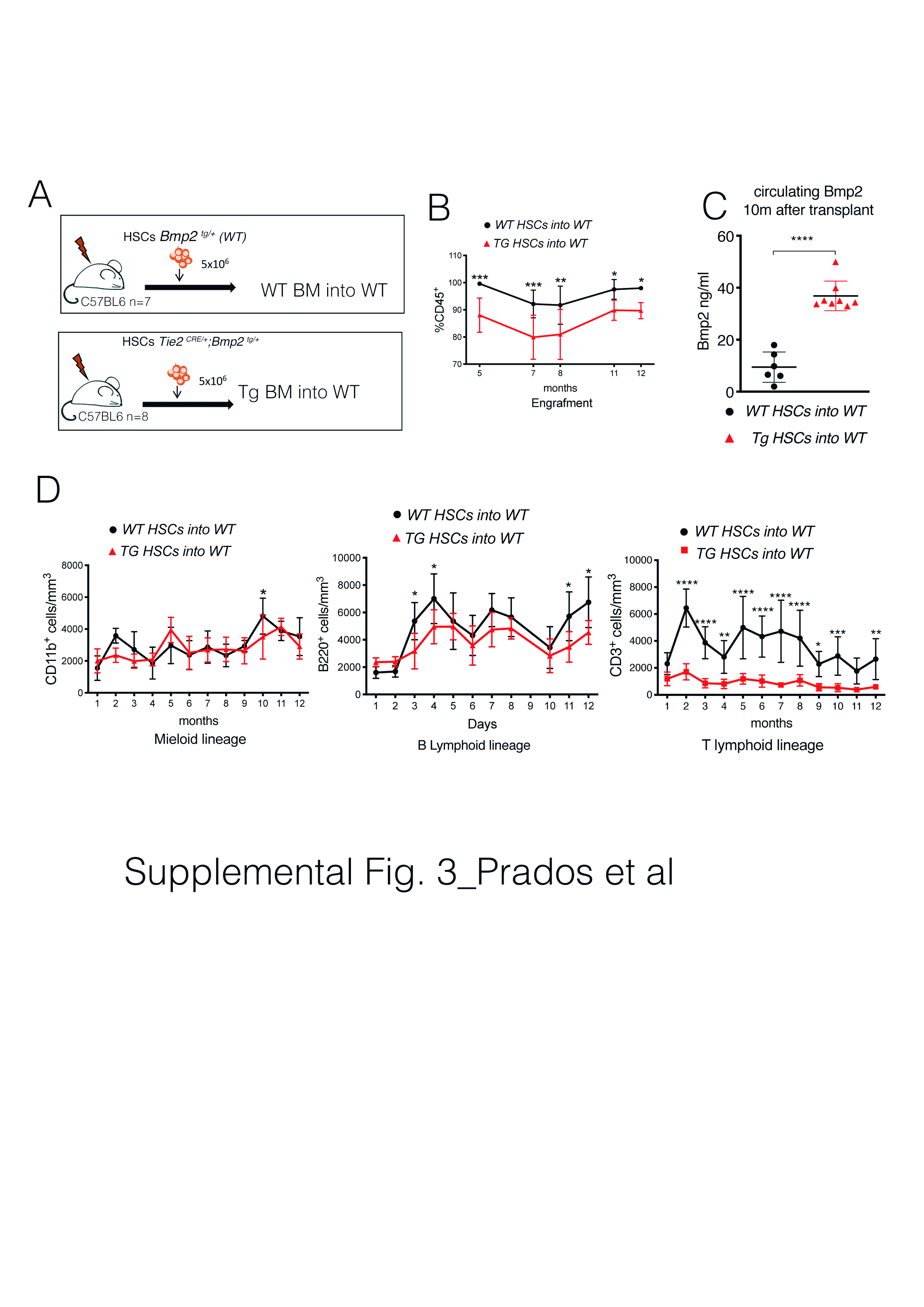

Supplement: Supplementary file 4 — Suppl. Figure 3 [file 41419_2021_4003_MOESM4_ESM.jpg]

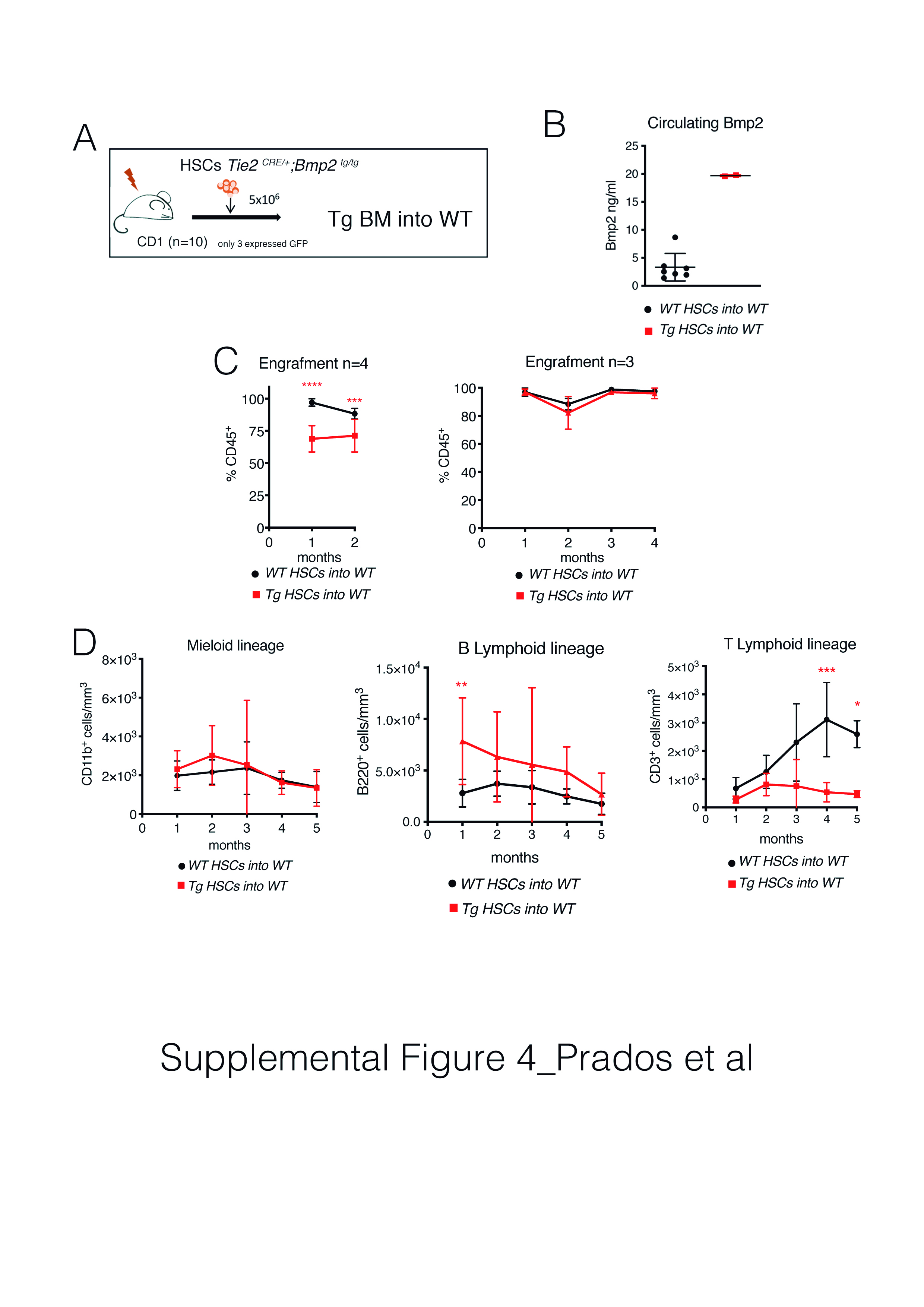

Supplement: Supplementary file 5 — Suppl. Figure 4 [file 41419_2021_4003_MOESM5_ESM.jpg]

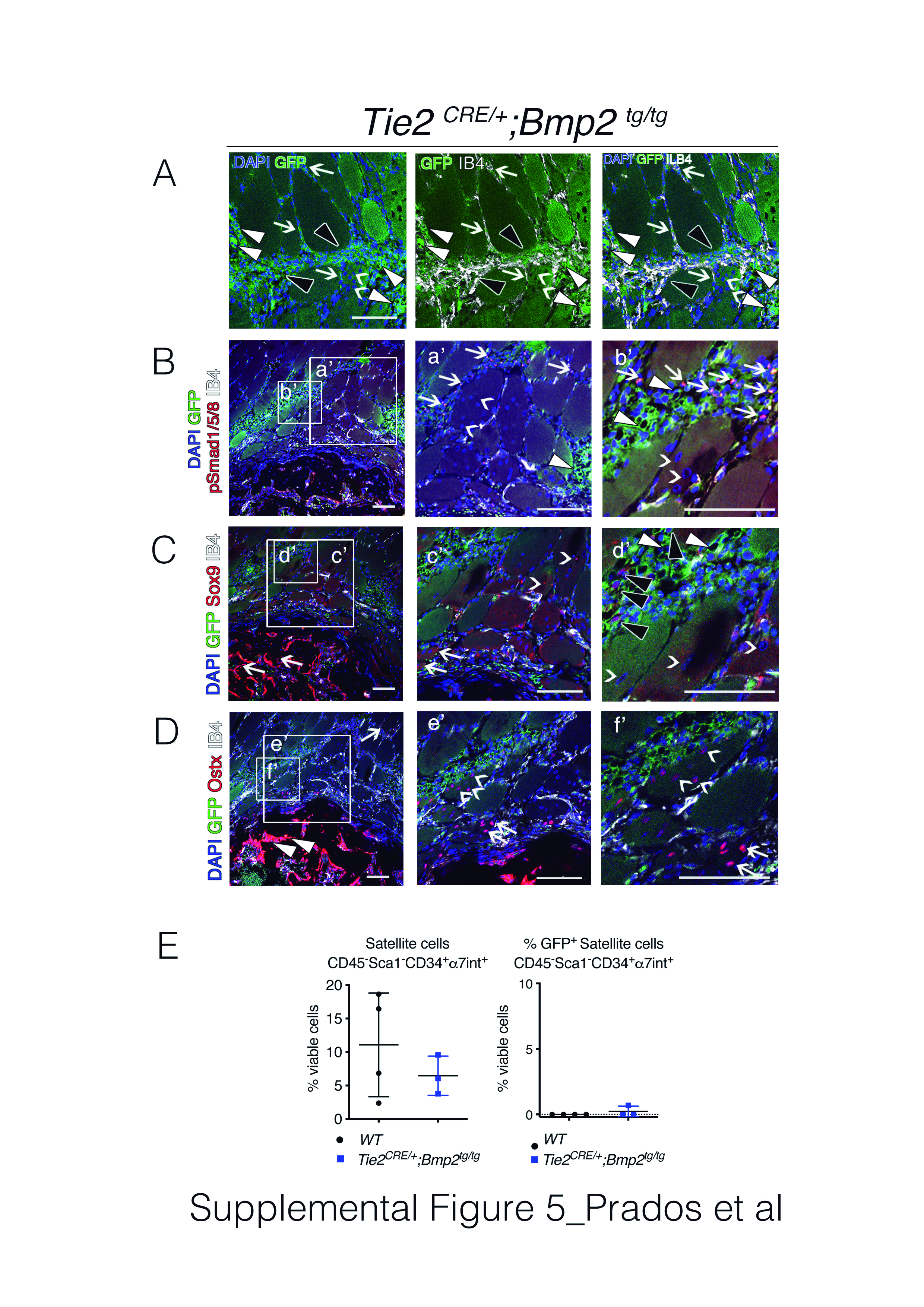

Supplement: Supplementary file 6 — Suppl. Figure 5 [file 41419_2021_4003_MOESM6_ESM.jpg]
